# Impact of anthropogenic activities on morphological and deposition flux changes in the Pearl River Estuary, China

**DOI:** 10.1038/s41598-021-96183-0

**Published:** 2021-08-17

**Authors:** Xing Wei, Shuqun Cai, Weikang Zhan

**Affiliations:** 1 State Key Laboratory of Tropical Oceanography, South China Sea Institute of Oceanology, Chinese Academy of Science, Guangzhou, 510301 China; 2 Southern Marine Science and Engineering Guangdong Laboratory (Guangzhou), Guangzhou, 511458 China; 3 Guangdong Key Lab of Ocean Remote Sensing, Guangzhou, 510301 China

**Keywords:** Environmental sciences, Ocean sciences, Solid Earth sciences

## Abstract

The evolution of the Pearl River Estuary (PRE), China in recent decades has been dominated by human activities. Historical admiralty charts and remote sensing images indicated that from 1936 to 2017, the tidal flat area and water area decreased by 23.6 × 10^7^ m^2^ and 60.7 × 10^7^ m^2^, respectively. The average advancing rate of the coastline of the PRE to the sea from 1972 to 2017 reached approximately 64.8 m/year, which is several times or even dozens of times that since the mid-Holocene. Land reclamation was the main reason for the dramatic changes in the water area and coastline. Although the water volume of the PRE showed a decreasing trend from 1936 to 2017, the water volume reduction rates for 1996–2005 and 2005–2017 were only 29% (1.27 × 10^7^ m^3^/year) and 12% (0.53 × 10^7^ m^3^/year), respectively, of that for 1936–1972. The combined influences of channel dredging, sand mining, and sediment load reduction caused by dam construction have contributed to this change. From the perspective of the filling up of the estuary, channel dredging, sand mining, and dam construction in the river basin are beneficial for prolonging the life of the estuary.

## Introduction

The estuary and its delta are the most concentrated areas of human activity in the world, with rich natural resources and an important geographical location. More than 500 million people live in the delta area^1^, which accounts for less than 1% of the global land area and features an economic output value of several trillions of dollars^2^. Therefore, the geomorphological evolution of the estuary and its delta are of great significance to the sustainable development of the region and the global economy.

Fluvial sediment is the main source of material for the construction of the estuaries. However, in recent decades, many large rivers around the world, such as the Nile^3,4^, Mississippi^5^, Mekong^6^, Ebro^7^, Yellow^8,9^, and Yangtze Rivers^10,11^, have experienced drastic reductions in sediment load delivered into the sea owing to dam construction, soil conservation and water diversion. In particular, it is estimated that reservoirs can effectively trap as much as ~ 5 billion tons per year of fluvial sediment, equating to approximately 25% of the global total sediment transport^12^. As a result, the estuaries of these rivers have been strongly eroded and their coastlines have quickly receded^8–10,13,14^. Meanwhile, human activities in estuaries, such as land reclamation, channel dredging and sand mining, can also cause dramatic changes in the topography of estuaries^15–18^. Thus, a better understanding of the changes in the estuary morphology and their response to human activities is of great significance for the environmental management of estuaries and their deltas.

**Figure 1:**
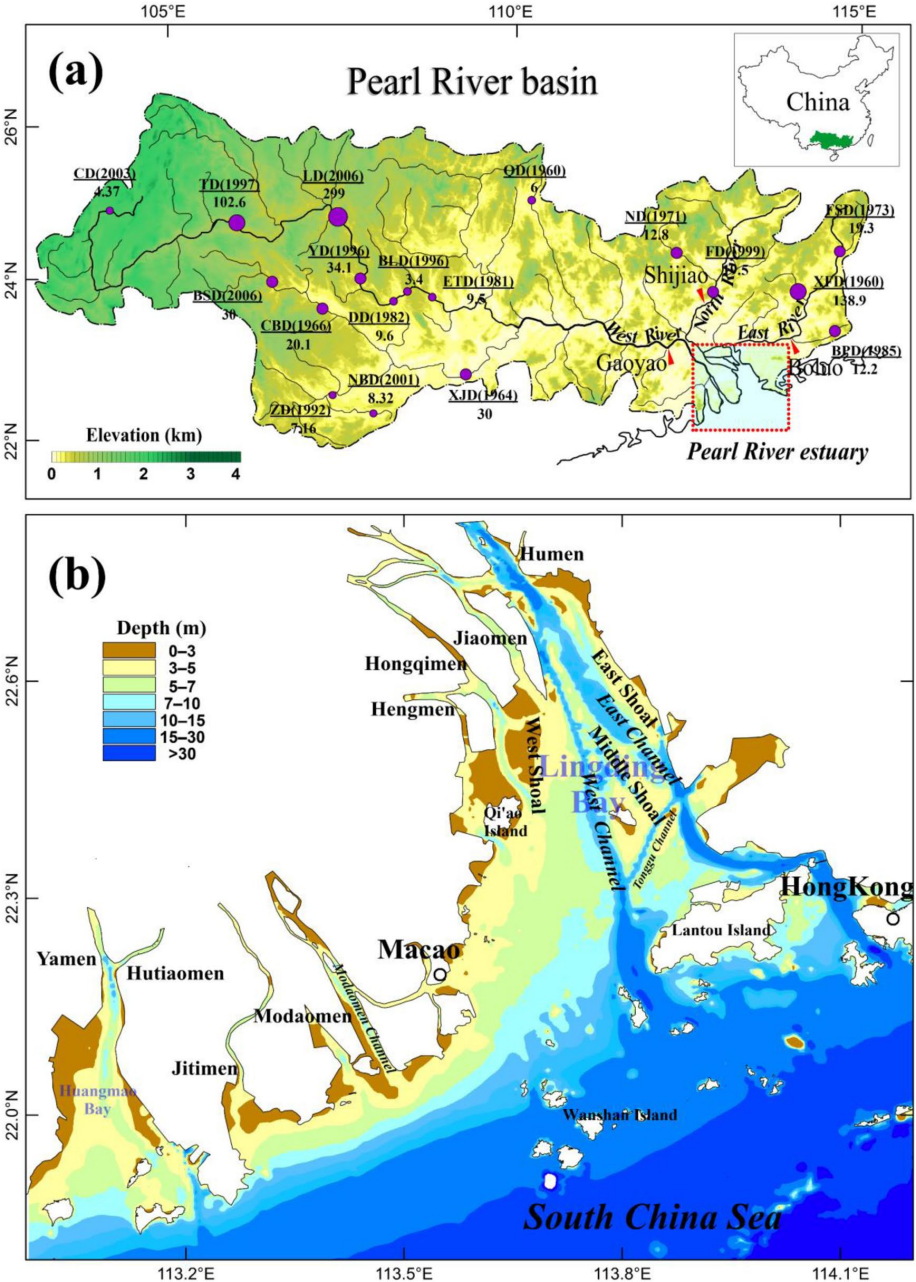
The Pearl River (**a**) and its Estuary (**b**). The index maps of the China and Pearl River basin were modified from Bulletins of Chinese sediment, https://www.mwr.gov.cn/sj/. The color scale of the relief map shows the land elevation above the sea level in meters, and the Digital Elevation Model (DEM) data are available at https://www.ngdc.noaa.gov/mgg/. Maps were created with Surfer, Version 13, https://www.goldensoftware.com/products/surfer, ArcGIS, Version 10.6, https://desktop.arcgis.com/zh-cn. Dam sites are denoted by circles with the name of the dam, year of dam closure in parentheses, and storage capacity of the corresponding reservoir (unit in × 10^8^ m^3^) under each name. The abbreviations of the dam names are as follows: CD (Caishitan dam), TD (Tianshengqiao dam), LD (Longtan dam), YD (Yantan dam), DD (Dahua dam), BLD (Bailongtan dam), ETD (Etan dam), BSD (Baise dam), CBD (Chengbihe dam), ZD (Zhuojiang dam), NBD (Nanshui dam), XJD (Xinjiang dam), QD (Qingshitun dam), ND (Nanshui dam), FD (Feilaixia dam), FSD (Fengshuba dam), XFD (Xinfengjiang dam), BPD (Baipenzhu dam).

The Pearl River is one of the world’s 25th largest rivers with respect to water discharge and sediment load^19^. Its estuary, the Pearl River Estuary (PRE), is located in Guangdong Province, South China (Fig. 1). It is one of the largest and most important estuaries in Asia. Before the 1980s, the evolution of the estuary was mainly dominated by natural processes because human activities based on the agricultural industry had little impact on the drainage basin and estuary. However, with the implementation of the national reform and opening policy in the 1980s, the population and economy of the Pearl River Basin began to develop rapidly. Human activities, including dam construction in the river basin^20–23^, land reclamation, channel dredging, and sand extraction in the estuary^24–26^, have greatly accelerated in the Pearl River Basin and the PRE. Consequently, the natural changes in the sediment load from the river and the morphological evolution of the estuary have been severely disturbed. Some studies have investigated the response of changes in the coastline and topography of the PRE to the reduction in sediment load caused by dam construction^27,28^ and land reclamation^26,29^. However, systematic research on the response of the morphological changes of the PRE to diverse human activities (e.g., land reclamation, channel dredging and sand mining) is still insufficient. The time range of the existing studies mostly covers the 1980s to the 2000s, and the spatial scope is limited to specific regions, such as Lingding Bay^30,31^, Modaomen sub-estuary^32,33^ and Huangmao Bay^34^, which are not sufficient to identify the detailed and holistic morphological changes in the PRE and to diagnose the impacts of diverse human activities. Moreover, the deposition flux is an important indicator parameter that reflects the evolution of the estuary and predicts its future development. However, there have been few reports on the changes in the deposition flux in the PRE and their response to human activities to date.

The Guangdong-Hong Kong-Macao Greater Bay Area (GBA) is one of the regions with the highest degree of openness and the strongest economic vitality in China. It also has an important strategic position in the development of the national economy. As the spatial carrier of the GBA, the PRE continues to be affected by the progress of the construction of the GBA. Therefore, environmental protection and sustainable development of the PRE will face highly severe challenges. In this study, a series of historical navigational charts (1936–2017) and satellite remote sensing maps (1973–2018) was compiled to explore the changes in the coastline, subaqueous topography and deposition flux of the PRE and the relationship between these changes and human activities in the drainage basin to the estuary. Conveniently, this time span of data provides us with a very different temporal perspective to compare the changes in morphological and sediment flux under the intervention of human activities of different intensities and to explore in detail the individual and comprehensive long-term effects of diverse human activities on the morphological evolution of the estuary. The PRE is a typical large-scale estuary comparable to many large estuaries globally, such as the Nile, Mekong, and Mississippi. The estuary dynamics and tidal flats are greatly influenced by the complex coastline composed of islands and so on, unlike some estuaries that are discharged directly into the open shelf. These local distinguishing features, together with others, make this study significant for improving our knowledge of the evolution of estuarine morphology. Moreover, this study also has important implications for understanding coastal environmental evolution mixed with human activities, providing further scientific guidelines for global river and estuary management.

## Results

### Changes in coastline

**Figure 2:**
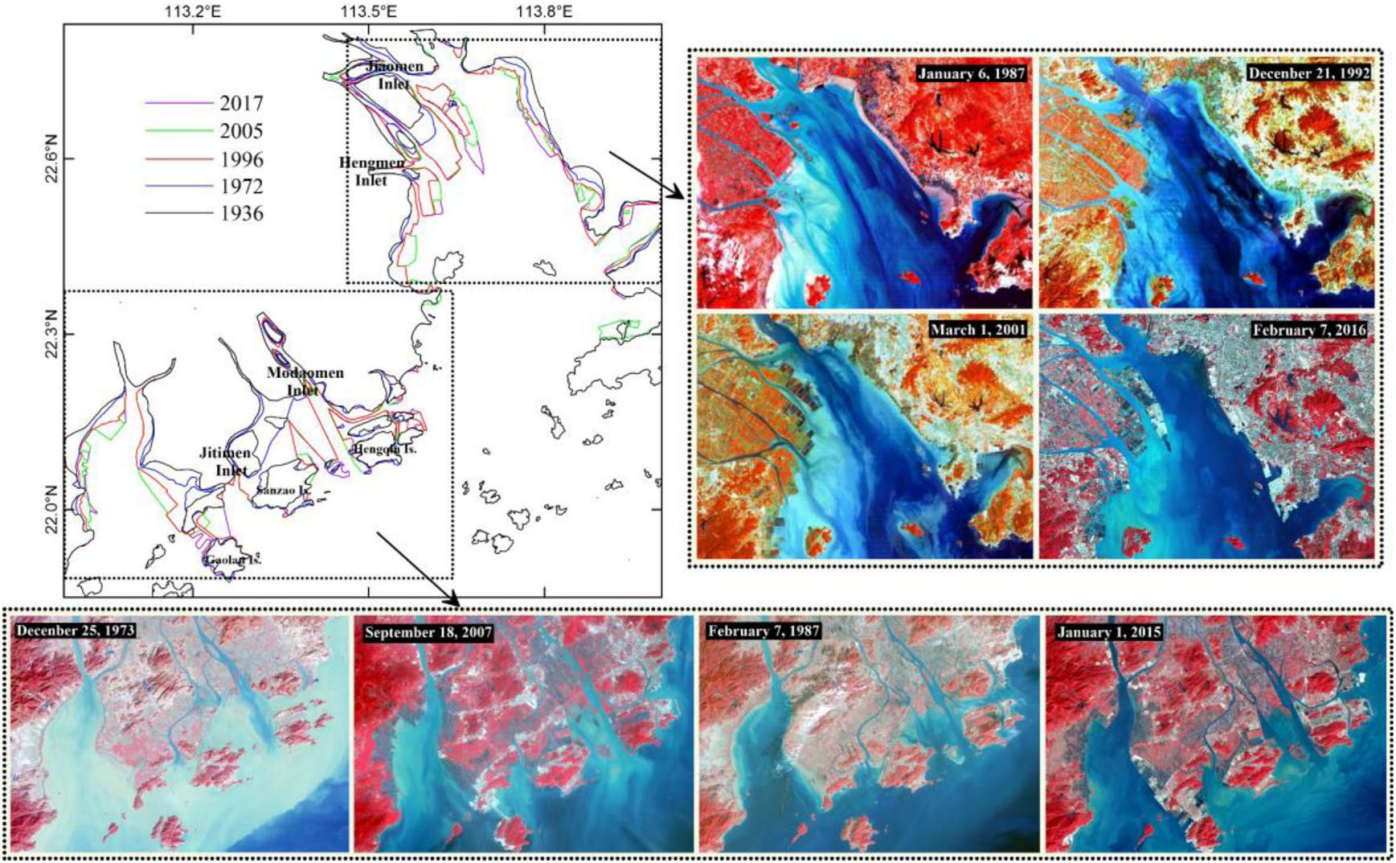
Coastline evolution in the Pearl River Estuary for 1936–2017 and comparison of the coastline of the sub-regions at different times. Maps were created with Surfer, Version 13, https://www.goldensoftware.com/products/surfer, ENVI, Version 5.3, http://www.enviidl.com/.

The coastline of the PRE changed significantly during 1936–2017 (Fig. 2). The length of the coastline in 2017 was 556.63 km, an increase of approximately 228 km from 1936 (Table 1). The spatial expansion of the coastline of the PRE can also be clearly identified from the satellite images. By comparing the images, it is clear that the inlets of the Pearl River Estuary have been continuously extended toward the sea. The areas with the most severe coastline extensions mainly occurred along the west coast of the Jiaomen, Modaomen, Hengmen, and Jitimen Inlets. Moreover, as the coastline extended, more than 20 near-shore islands, such as the Gaolan, Sanzao, and Hengqin Islands, gradually merged into the mainland. This extension of the coastline has caused a continuous decrease in the overall area of tidal flats and water in the estuary. As shown in Table 2, from 1936 to 2017, the tidal flat area and water area decreased by 23.6 × 10^7^ m^2^ and 60.7 × 10^7^ m^2^, respectively. By contrast, the corresponding land area increased by 84.3 × 10^7^ m^2^. Lingding Bay, a trumpet-shaped sub-estuary of the PRE, receives approximately half of the river discharge through the four easternmost inlets: Human, Jiaomen, Hongqimen, and Hengmen (Fig. 1). During 1936–2017, the Lingding Bay coastline extended by approximately 88 km, while the western coast of Lingding Bay advanced approximately 10 km from the sea. Corresponding to the growth of the coastline, the water area of Lingding Bay decreased by 276 km^2^, which is 28.6% of the current area of Lingding Bay.

**Table 1:** Coastline length, tidal flat area, subaqueous area and water volume in the Pearl River Estuary, 1936–2017.

Year	1936	1972	1996	2005	2017
Coastline length (km)	328.56	378.60	475.00	518.24	556.63
Tidal flat area (km^2^)	489.5	576.9	378.6	266.9	253.2
**Subaqueous area (km**^**2**^**)**					
0–2 m	76.14	71.79	109.01	157.06	178.28
2–5 m	988.59	1019.28	943.55	971.56	1004.89
5–10 m	1674.32	1432.58	1204.01	1082.44	944.48
> 10 m	263.15	201.55	207.73	222.24	268.05
Subtotal	3002.2	2725.2	2464.3	2433.3	2395.7
Mean water depth (m)	6.27	6.06	5.92	5.91	5.97
Water volume (km^3^)	19.77	18.16	17.16	17.03	16.96

**Table 2:** Changes in coastline, tidal flat area, water area, land area and water volume in the estuary for different time periods.

Period	Coastline extension		Tidal flat area loss		Water area loss		Land area gain		Water volume loss	
	Total (10^4^ m)	Rate (10^3^ m/year)	Total (10^7^ m^2^)	Rate (10^6^ m^2^/year)	Total (10^7^m^2^)	Rate (10^6^ m^2^/year)	Total (10^7^ m^2^)	Rate (10^6^ m^2^/year)	Total (10^9^ m^3^)	Rate (10^7^ m^3^/year)
1936–1972	5.0	1.4	− 8.7	− 2.4	27.7	7.7	19.0	5.3	1.74	4.35
1972–1996	9.6	4.0	19.8	8.3	26.1	10.9	45.9	19.1	1.03	4.00
1996–2005	4.3	4.8	11.2	12.4	3.1	3.4	14.3	15.9	0.14	1.27
2005–2017	3.8	3.1	13.7	1.1	3.8	3.1	18.8	15.7	0.08	0.53
1936–2017	22.8	2.8	23.6	2.9	60.7	7.5	98.0	12.1	2.81	3.42

**Figure 3:**
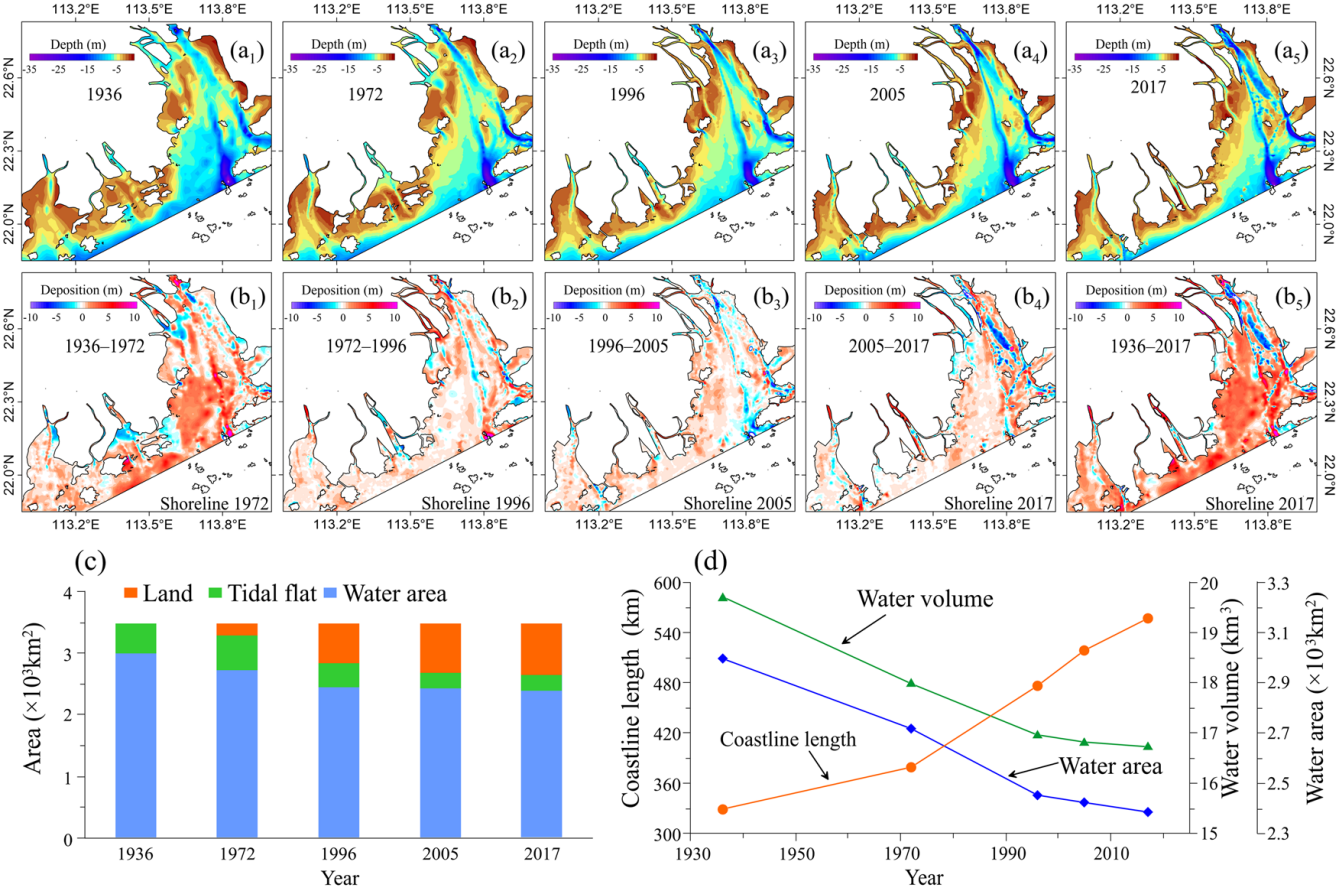
Bathymetric maps of the Pearl River Estuary in 1936, 1972, 1996, 2005, 2017 (**a**) and bathymetric change maps during different periods (**b**). Changes in land area, tidal flat area, and water area for 1936–2017 (**c**). Total length of coastline, water area, and estuary volume for 1936–2017 (**d**). Maps created with Surfer, Version 13, https://www.goldensoftware.com/products/surfer, Histograms and line charts were created with Microsoft Office, Version 2016, https://www.microsoft.com/zh-cn/.

Additionally, from the image comparison it can also be clearly identified that the extent and intensity of coastline changes in different periods are significantly different. From 1936 to 1972, the coastline slowly increased at a rate of 1.4 km/year. However, during 1972–1996 and 1996–2005, the coastline increased rapidly at 4.0 km/year and 4.8 km/year, respectively. From 2005 to 2017, the rate of coastline extension slowed down significantly compared to that of the other two time periods. It was also found that the decrease in tidal flat and water areas often corresponded to an increase in land (Fig. 3c). The fastest water area loss occurred during 1972–1996, at a rate of 10.9 × 10^7^ m^2^/year.

### Changes in subaqueous topography

From 1936 to 2017, the subaqueous topography of the estuary experienced drastic changes, and the contours continued to move towards the sea (Fig. 3a). The − 5 m isobath migrated to the sea at an average distance of 5 km. In particular, on the west side of Lingding Bay the maximum distance of the − 5 m isobath propulsion to the sea from 1936 to 2017 could reach a maximum of 15 km.

Most areas of the PRE were in a state of siltation during 1936–2017 (Fig. 3b), which accounted for 79.88% of the total area of the estuary (Table 3). The mean water depth of the estuary continuously decreased from 6.27 m in 1936 to 5.97 m in 2017. Correspondingly, the water volume of the estuary decreased from 19.77 km^3^ in 1936 to 16.96 km^3^ in 2017, and there was a total reduction of 2.81 km^3^ over the 81-year period. However, the rate of water volume loss in the estuary showed a decreasing trend since 1936 (Fig. 3d). The rates of water volume loss in 1936–1972 and 1972–1996 were 4.35 × 10^7^ m^3^/year and 4.00 × 10^7^ m^3^/year, respectively, while for 1996–2005 and 2005–2017, were 1.27 × 10^7^ m^3^/year and 0.53 × 10^7^ m^3^/year, respectively.

**Table 3:** Changes in erosion and depositional areas in the estuary in different periods.

Period	1936–1972	1972–1996	1996–2005	2005–2017	1936–2017
Erosion area (km^2^)	681.2	633.7	745.0	713.2	469.1
Percentage of erosion area (%)	25.00	25.72	30.62	29.77	19.58
Erosion rate (cm/year)	− 2.21	− 2.84	− 6.51	− 6.67	− 5.01
Depositional area (km^2^)	2028	1810.6	1674.3	1669.5	1913.6
Percentage of depositional area (%)	74.42	73.47	68.81	69.69	79.88
Deposition rate (cm/year)	4.32	4.58	3.84	3.73	4.13
Total bathymetry change rate (cm/year)	2.18	2.09	1.12	1.07	1.44

### Changes in deposition flux

In the PRE, the mean deposition thickness in the depositional area was between 2 and 3 m. The deposition rate in the depositional area was 4.32 cm/year for 1936–1972, and it increased to 4.58 cm/year in 1972–1996. Since 1996, the deposition rate decreased (Table 3). The deposition rates for 1996–2005 and 2005–2017 were 3.84 cm/year and 3.73 cm/year, respectively. Erosion areas in 1936–1972 and 1972–1996 were 681.2 km^2^ and 633.7 km^2^, respectively, and increased to 745.0 km^2^ for 1996–2005. For 2005–2017, the erosion areas became 713.2 km^2^, a decrease of 31.8 km^2^ compared to that in 1996–2005. The proportions of eroded area in the four periods were 25.00%, 25.72%, 30.62% and 29.77%, respectively. Erosion rates in the erosion areas were − 2.21 cm/year and − 2.84 cm/year for 1936–1972 and 1972–1996, respectively. For 1996–2005 and 2005–2017, rates exceeded 6.5 cm/year, more than twice the previous values. As shown in Fig. 3b, the erosion areas were mainly distributed in navigation channels and island-forming fjord areas, such as the Chuanbi Channel, Longgu Channel, and Lantau Strait in Lingding Bay.

The mean annual deposition flux of the PRE from 1936 to 2017 was approximately 32.99 Mt/year, and it showed a decreasing trend (Table 4). In 1936–1972, the mean annual deposition flux was 56.52 Mt/year, and it dropped slightly to 52.04 Mt/year in 1972–1996. However, in 1996–2005 and 2005–2017, the mean annual deposition flux dropped sharply to 16.47 Mt/year and 6.92 Mt/year, respectively, which were only 29.1% and 12.2% of that in 1936–1972.

**Table 4:** Changes of deposition flux and sediment load in the estuary in different periods.

Period	1936–1972	1972–1996	1996–2005	2005–2017	1936–2017
Sediment load s_0_ (Mt/year)	78.51	82.63	49.95	24.73	58.96
Deposition flux d_0_ (Mt/year)	56.52	52.04	16.47	6.92	32.99
Capture ratio (Δ = d_0_/s_0_)	0.72	0.63	0.66	0.28	0.56

Values were determined using a dry bulk density of 1.1 kg/m^3^.

## Discussion

From the first principles, sea level rise will create an area of potential submergence and an accommodation volume for sediments to fill. The degree of estuary progradation thus depends on the rate of accommodation increase versus the rate of sediment supply. From the past century, the sea level worldwide has been rising owing to global warming. It was suggested that the relative mean sea level in the PRE has risen at a rate of about 2.3 mm/year over the past few decades, reflecting the combined effects of changes in mean eustatic sea level, tidal range, and vertical land movements^35,36^. If continued for any significant time period, sea level rise might be expected to cause the estuary to retreat landward. However, the total magnitude of recorded sea-level rise for 1936–2017 (ca. 18.6 cm) is unlikely to have played a significant role in determining the major changes in the coastline and water volume of the PRE. As shown in Fig. 3 and Table 3, the topographical changes of the PRE in recent decades are mainly reflected in the following features: (1) the accelerated advancement of the coastline toward the sea, (2) the sharp decrease in deposition flux, and (3) the severe downcutting of the seabed. The timescale and magnitudes of these changes show no identifiable correlation with the recorded changes in mean sea level. They are mainly caused by the massive intervention of diverse human activities.

**Figure 4:**
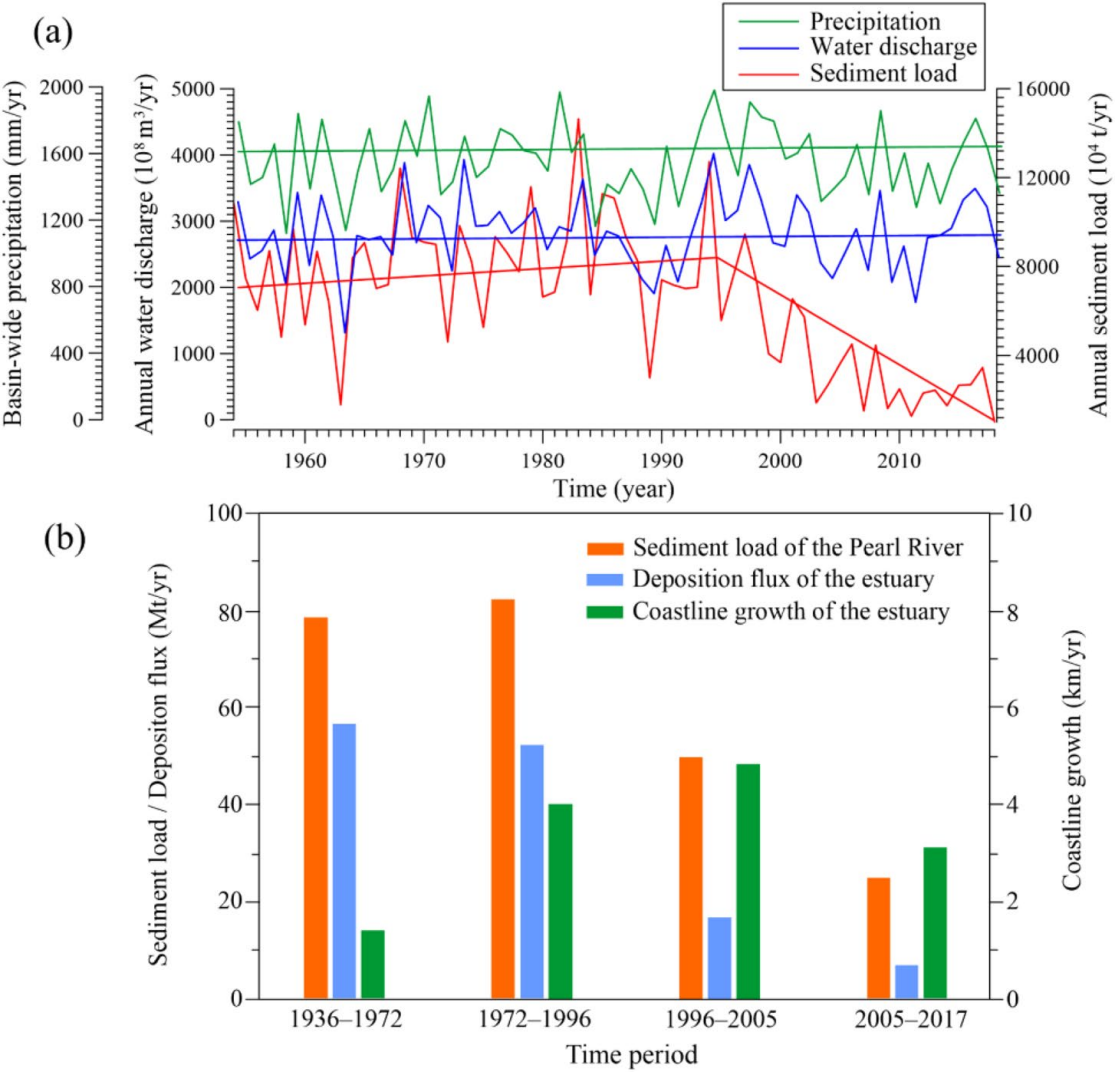
Annual precipitation, water discharge and sediment load of the Pearl River from 1954 to 2018 (**a**), and relationship between the sediment load from the Pearl River and the growth of the coastline and deposition flux of the estuary in different periods (**b**). Histograms and line charts created with Grapher, Version 2016, https://www.goldensoftware.com/products/grapher.

Second, according to hydrological data from 1954 to 2018, there were no significant decreasing trends in the precipitation or water discharge time series (Fig. 4a). However, the sediment load exhibited a significant decreasing trend since the 1990s, regardless of the water discharge. Dam construction in the drainage basin is the main reason for the drastic reduction in sediment load^20,22,23,37^. Statistics indicate that over 9000 dams and reservoirs have been constructed in the Pearl River Basin since the 1950s. The total storage capacity of reservoirs was estimated to be approximately 75 × 10^9^ m^3^ in the 2010s, 27% of the multi-annual mean discharge of the Pearl River. This value can be compared to the findings of Vörösmarty et al.^12^, who determined that large reservoirs intercept more than 40% of the global river discharge. Figure 1a presents the locations of the primary large reservoirs (i.e., storage capacities exceeding 10^8^ m^3^) spread across the Pearl River Basin. From the 1960s to the 1990s, the total storage capacity of the Pearl River Basin increased slowly, followed by a boom in dam reservoir construction since the 1990s^37^. Based on this change, the sediment load decreased significantly since the late 1990s, as large amounts of sediment became trapped inside reservoirs (Fig. 4a). In 1999, the sediment load decreased by one-third of the level of 1980s following the construction of the Tianshegnqiao dam (TD), Yintan dam (YD), and Bailongtan dam (BLD). In 2007, the sediment load decreased to 15.08 × 10^4^ t/year, which is approximately 19% of the 1954–1979 reference level, based on the construction of the Baise dam (BSD) and Longtan dam (LD). It should be noted that LD, the largest dam in the Pearl River Basin, was constructed in 2006 with a storage capacity of 29.9 × 10^9^ m^3^. According to the Mann–Kendall test analysis, Wei et al. (2020) suggested that the abrupt change in sediment load during this period occurred in 1998^23^. The linear regression equations for annual sediment load suggest a decrease of 178 × 10^4^ t/year from 1998 to 2018. Because the sediment load by rivers is the main source of material deposition in the PRE, the drastic decrease in sediment load has had an important impact on the evolution of the estuary. As shown in Fig. 4b, the sediment load is significantly correlated with the deposition flux of the estuary. Before 1996, the annual sediment load was ~ 80 Mt/year, and the corresponding deposition flux at the estuary was ~ 54 Mt/year. Subsequently, the annual sediment load decreased to ~ 37 Mt/year, less than half of the previous amount. Correspondingly, the deposition flux of the estuary decreased to ~ 12 Mt/year. However, for the current evolution of the PRE, the coastline has shown a rapid growth trend owing to reclamation. The impact of the reduction of sediment load on the forward siltation of the estuary is not yet clear. However, for the future development of the estuary, the reduction in sediment load will inevitably slow down the rate of delta progradation. Moreover, the reduction in the sediment load also disrupted the existing sedimentary balance in the PRE, and some areas that were originally in a sedimentation equilibrium state were eroded owing to insufficient sediment supply. According to the DEM data, the area and rate of erosion in the PRE showed an increasing trend (Table 3). During 1996–2005 and 2005–2017, the erosion areas were 113.3 km^2^ and 79.5 km^2^, respectively, larger than that for 1972–1996. The erosion rate increased from 2.21 cm/year for 1936–1972 to 6.67 cm/year for 2005–2017. Correspondingly, the percentage of depositional area of the estuary decreased from 73.47% for 1972–1996 to 69.69% for 2005–2017, and the deposition rate decreased from 4.58 cm/year for 1972–1996 to 3.73 cm/year for 2005–2017. The decrease in sediment load is an important contribution to this change. A similar response of decreased sedimentation to human activities has been reported for different estuaries^17,18,38^. In the future, with increased dam construction and further intensification of the afforestation policy in the greater drainage basin, the sediment load of the Pearl River is expected to decrease further, causing a further increase in the erosion area of the estuary.

**Figure 5:**
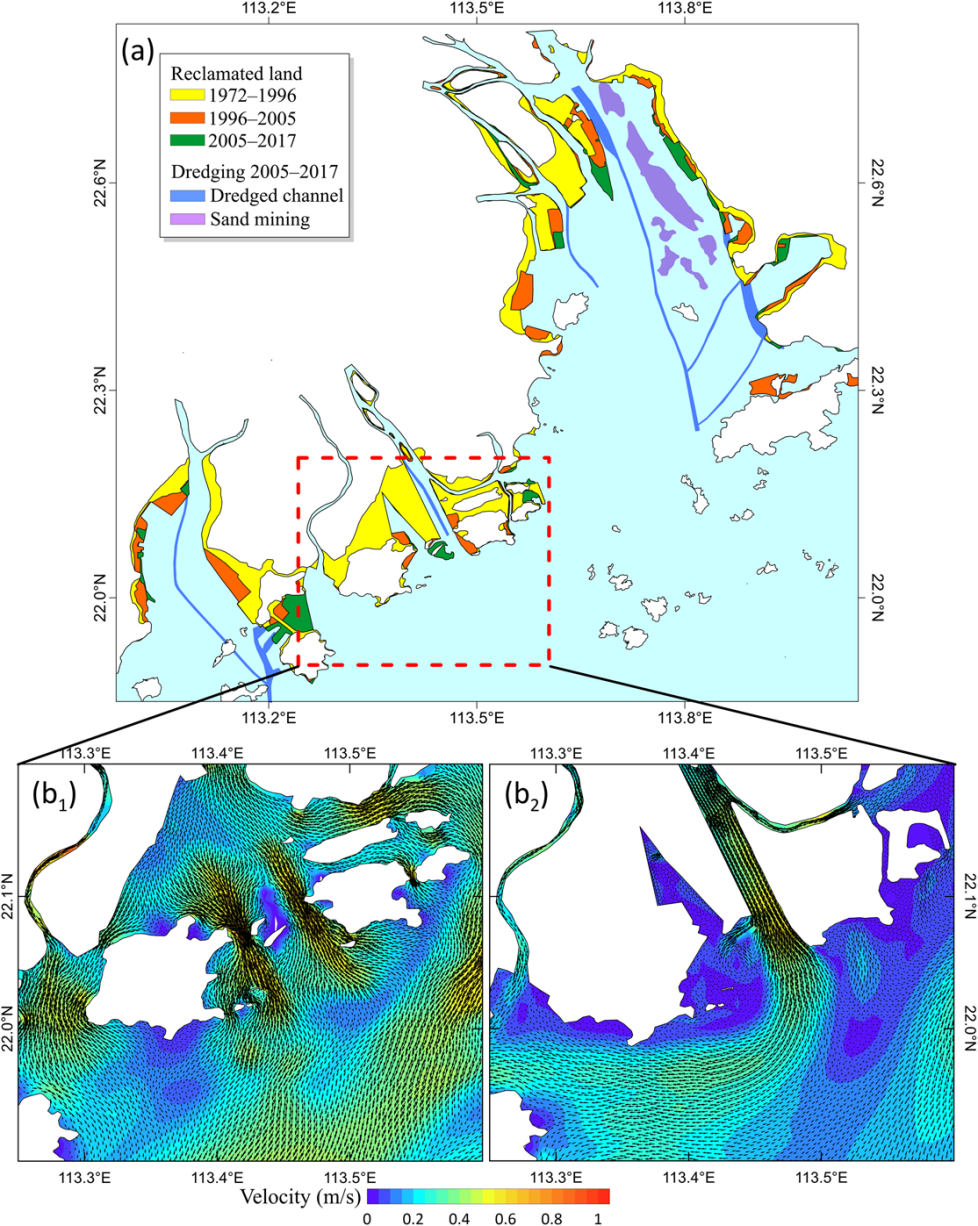
Schematic diagram of the locations of land reclamation, dredging channels, and sand mining in the Pearl River Estuary for 1972–2017 (**a**), and the ebb tide flow field at the Modaomen sub-Estuary in the summer of 1977 (**b**_**1**_) and 2003 (**b**_**2**_). Maps were created with Surfer, Version 13, https://www.goldensoftware.com/products/surfer.

**Figure 6:**
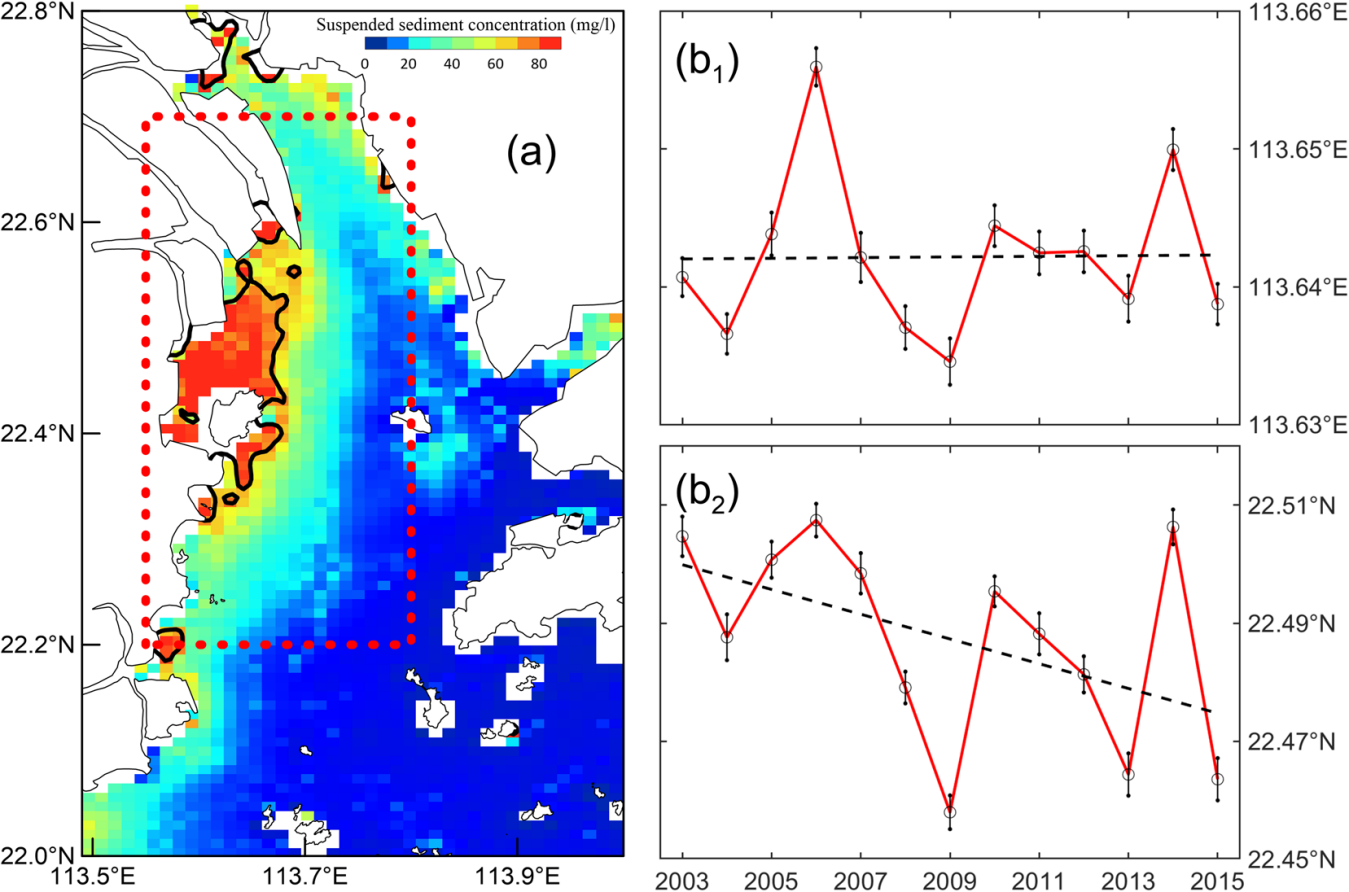
The distribution of SSC and the location of the zone where the quantile of SSC was greater than 90th in Lingding Bay of PRE in the wet season of 2015 (**a**). The Migration trend of this zone in the longitude and latitude directions from 2003 to 2015 (**b**). The black bold solid line in (**a**) represents the 90th isoline of the quantile of SSC. The black circles, error bars and dashed lines in (**b**) represents the annual wet season SSC, the corresponding 95% confidence interval and the linear regression trend, respectively. It can be summarized from (**b**) that the maximum SSC zone of the Lingding Bay did not generally shift westward during 2003–2015, but moved about 2.3 km to the south. Maps were created with Matlab, Version R2014, https://ww2.mathworks.cn/.

Third, with the rapid economic development and the explosive increase in population in the Pearl River Delta region, land reclamation activities have increased dramatically to ease the increasing land demand^26^. From 1972 to 2017, a total of 657 km^2^ of land was reclaimed (Fig. 5a), and the average coastline advancing rate toward the sea reached approximately 64.8 m/year. In particular, this rate could even reach approximately 256.3 m/year if only Lingding Bay was considered. Research shows that the modern Pearl River Delta was formed by the filling of sediments from the Pearl River after the sea level rose to relative stability during the mid-Holocene^39^. Since 6000 year BP (before present), the coastline of the Pearl River Estuary has been advancing toward the sea at an average rate of about 16 m/year^40^. This demonstrates that the coastline advancement caused by land reclamation has far exceeded the natural accumulation process. The natural evolution of the PRE has been profoundly affected by land reclamation. First, the bifurcation and extension of river channels have also been restricted by land reclamation. Generally, when a river discharges into a sea, the flow diverges and results in mouth-bar deposition. The progradation of the coastline leads to bifurcation of the river and the creation of a new distributary. However, as a result of land reclamation, the river loses accommodation for bifurcation. Considering the Modaomen outlet as an example, land reclamation has caused a sharp reduction in the water area of the estuary, and the Modaomen Channel developed straight to the sea under the control of the artificial dike (Fig. 5b). Simultaneously, the rapid extension of the outlets caused the sediment from the rivers to be transported further downstream of the estuary (Fig. 6), which in turn promoted the rapid downstream movement of the maximum turbidity zone and deposition center. It has been reported that the deposition center of Lingding Bay in 2016 was mainly located in the south of Qi’ao Island, which has moved downstream by nearly 17 km from 1975^31^.

Fourth, shipping in the PRE is highly developed, and its port throughput accounts for one-quarter of the shipping activity in China. However, the waterways of these ports are continuously back-silting so that dredging is required several times a year to ensure continued shipping activity. Affected by channel dredging, the navigation channels gradually became narrower and deeper (Fig. 3a). For example, in the west channel of Lingding Bay, the water depth increased from 6.8–8.5 m in 1974 to 9.1–10.5 m in 1989, 9.8–12.0 m in 1998, and 11.5–13.5 m in 2000 owing to the channel dredging. Currently, the total length of the channel to be dredged in the PRE is approximately 200 km, occupying an area of approximately 70 km^2^ (Fig. 5a). Additionally, sand excavation also contributed to the downcutting of the estuary. According to the DEM data, the average water depth of the sand mining area of Lingding Bay from 1972 to 2017 increased by approximately 2 m. The most serious area of sand mining is located in the middle shoal. Sand excavation caused the seabed in this area to be generally downcut by 5–10 m, and the maximum downcut reached 17 m. Many deep potholes of different sizes are formed on the seabed. Therefore, channel dredging and sand excavation have a significant impact on the underwater topography of estuaries. The seabed topography will be in the process of self-adjustment for a long time in the context of a drastic decrease in sediment load.

In general, the combined influence of sediment load reduction and land reclamation caused the water volume of the PRE to show a decreasing trend from 1936 to 2017. However, the rate of decrease in the water volume in 1996–2017 was significantly lower than that in 1936–1996. Notably, in 2005–2017, the mean water depth of the estuary showed a slight increase (Table 1), and the sediment capture rate of the estuary plummeted from 0.66 in 1996–2005 to 0.28 (Table 4). The reduction in sediment load, especially caused by channel dredging and sand mining in the estuary, contributed to these changes. According to Wu et al., during 1980–2015, channel dredging and sand mining removed nearly 3.6 Mt/year of sediment from upper Lingding Bay, which is equivalent to 30% of the total sediment load during this period^30^. Therefore, from the perspective of increasing water volume, channel dredging and sand mining are also beneficial for prolonging the life of the PRE. Careful and reasonable planning should be carried out before channel dredging and sand mining to play a beneficial role. Other human activities, such as constructing of the dump of marine garbage, the Hong Kong-Zhuhai-Macao Bridge, and aquaculture in marine cages, have also influenced the local topography and hydrodynamics of the estuary^25,41^. However, comparted with the impacts of land reclamation, channel dredging, and sand mining, the impacts of these other human activities are insignificant. With the further advancement of economic development in the Guangdong-Hong Kong-Macao GBA, the PRE will face even more severe challenges in estuary management and ecological and environmental protection^42–45^. Therefore, to predict possible future changes in estuary morphology, sedimentary characteristics, and water quality and associated impacts on biota and human usage of estuaries, further research should be carried out.

Worldwide, sediment supply reduction using dams, land reclamation, channel dredging, and sand mining are common practices in many estuaries. This study showed that the impact of such work on estuarine morphodynamics may be substantial and prolonged. Combining historical bathymetric charts, topographical survey data, and remote sensing data for comparative analysis is an effective method for picturing intermediate (decadal to centennial) timescale changes in estuary morphology and evaluating the main causes of these changes^6,15,46^. However, to quantify the impact of individual human activities on the hydrodynamics and sedimentation of the estuary and its long-term effects, numerical modeling will be needed in future studies.

## Conclusion

The evolution of the PRE has been intensely disturbed by diverse human activities in recent decades. Owing to land reclamation, the tidal flat area and water area decreased by 23.6 × 10^7^ m^2^ and 60.7 × 10^7^ m^2^, respectively from 1936 to 2017. The maximum loss rate of the water area occurred during 1972–1996, which was 10.9 × 10^6^ m^2^/year. The average advancing rate of the coastline of the PRE to the sea from 1972 to 2017 reached approximately 64.8 m/year, which is several times or even dozens of times that since the mid-Holocene. This change in turn caused a rapid downstream shift in the maximum turbidity zone and deposition center of the estuary. The bifurcation and extension of river channels have also been restricted by land reclamation. From 1936 to 2017, the water volume and deposition flux of the PRE showed a decreasing trend. However, the rate of decrease in water volume and deposition flux has dropped sharply since the 1990s. The combined influences of channel dredging, sand mining, and sediment load reduction caused by dam construction have contributed to this change. On the one hand, a large amount of sediment was moved away from the estuary owing to channel dredging and sand mining, leading to a decrease in the rate of sediment trapping by the estuary and severe downcutting of the seabed. On the other hand, the drastic reduction in sediment load caused by dam construction has disrupted the balance of sediment in the estuary, leading to an increase in estuary erosion. Furthermore, the inadequate supply of sediment in the estuary prolonged the natural restoration of the seabed. From the perspective of the filling up of the estuary, channel dredging, sand mining, and dam construction in the river basin are conducive to extending the life of the estuary.

## Data and methods

### Admiralty chart data

Historical admiralty charts were collected to help quantify the morphological changes in the study area. The scale of the charts ranged from 1:50,000 to 1:200,000 with a data density of 8–20 points per km^2^. The chart surveys were mainly carried out during five periods: the 1930s, 1960s, 1980–1990s, 2000s, and the 2010s, while the charts for each period generally consisted of several surveys. First, seven admiralty charts were transformed into depth points relative to the UTM49N-WGS84 coordinates using ArcInfo in a geographical information system (GIS). Then, the bathymetric data from each survey were gridded using the Kriging scheme into a 50 × 50 m resolution to produce an underwater digital elevation model (DEM) by GIS software^47,48^. Finally, the isobath evolution, scour, and silting developments of the PRE were obtained by subtracting the two subsequent morphological surveys.

### Satellite data

Satellite images from 1973 to 2018 were collected as additional information for analyzing the changes in the coastline of the PRE. The spatial resolution of the TM, ETM+ and OLI/TIRS images was 30 m and of the MSS images was 80 m. First, to match the images’ geographical reference coordinates of WGS84 of China, the column and line locations of pixels in the images were transformed by a second-order polynomial. Atmospheric correction was then performed using the haze reduction method. Finally, using stretching image enhancement techniques, the mean high tide lines of the false color composite images were extracted to serve as indicators of the coastline^49^.

Suspended sediment concentration (SSC) data in the PRE were derived from the Moderate-Resolution Imaging Spectroradiometer (MODIS) primary product (https://earthdata.nasa.gov/about/daacs/daac-laads). These data have a spatial resolution of 1 km and a temporal resolution of 1 d and are available for the period from January 1, 2003, to December 31, 2015. MODIS primary product processing includes two main aspects: atmospheric correction and SSC inversion. Therefore, SeaDAS 7.4 was used to select the shortwave infrared exponential algorithm^50^ for atmospheric correction of MODIS primary product data. The dual-band empirical model^51^ was then used to invert the SSC. Additionaly, the quantile regression analysis method was used to calculate the migration distance of the maximum SSC zone in Lingding Bay. A more detailed calculation process can be found in Zhan^52^.

### Numerical model data

A validated hydrodynamic model^53^ was used to investigate the dynamic response of the PRE to topography changes. The model adopts the Semi-implicit Eulerian–Lagrangian Finite Element (SELFE) model and has been widely used to study coastal and estuarine hydrodynamics^54,55^. Relevant details about the numerical technique implementations and validation can be found in Ni et al.^53^.
